# Grasping knowledge, attitude, and perception towards monkeypox among healthcare workers and medical students: an Egyptian cross-sectional study

**DOI:** 10.3389/fcimb.2024.1339352

**Published:** 2024-02-12

**Authors:** Fatma A. Amer, Hanaa A. Nofal, Manar G. Gebriel, Aya M. Bedawy, Ayman A. Allam, Hend E. S. Khalil, Mohammed Elahmady, Hagar Nofal, Maysaa A. Saeed, Shaker Wagih Shaltout, Ahmed Behiry, Osama Attia, Shereen Mohamed Bahgat, Ahmed A. Ali, Fatma Mohammed Ahmed, Ahmed Mohammed Abdelrahman, Noha M. Hammad

**Affiliations:** ^1^ Department of Medical Microbiology and Immunology, Faculty of Medicine, Zagazig University, Zagazig, Egypt; ^2^ Viral Infection Working Group of International Society of Antimicrobial Chemotherapy (VIWG/ISAC), London, United Kingdom; ^3^ Department of Public Health and Community Medicine, Faculty of Medicine, Zagazig University, Zagazig, Egypt; ^4^ Microbiology and Immunology, Qatar Armed Forces Hospital, Doha, Qatar; ^5^ Microbiology, Al Ahli Hospital, Doha, Qatar; ^6^ Department of Dermatology, Venereology and Andrology, Faculty of Medicine, Zagazig University, Zagazig, Egypt; ^7^ Department of Tropical Medicine, Zagazig University, Zagazig, Egypt; ^8^ Department of Tropical Medicine, Port Said University, Port Said, Egypt; ^9^ Department of Internal Medicine, Faculty of Medicine, Zagazig University, Zagazig, Egypt; ^10^ Department of Family Medicine, Faculty of Medicine, Zagazig University, Zagazig, Egypt; ^11^ Department of Pediatrics, Faculty of Medicine, Zagazig University, Zagazig, Egypt; ^12^ Department of Community Health Nursing, Faculty of Nursing, Zagazig University, Zagazig, Egypt; ^13^ Undergraduate Student, Faculty of Medicine, Zagazig University, Zagazig, Egypt

**Keywords:** Egypt, healthcare workers, KAP study, medical students, Mpox

## Abstract

**Background:**

Monkeypox (Mpox) is a re-emerging infectious disease representing a new global challenge. It poses a substantial threat to countries, particularly those with a low number of cases. Due to its popularity as a tourist destination and its proximity to many African refugees, Egypt is potentially at risk of Mpox importation. Therefore, effective disease management necessitates healthcare workers (HCWs) to possess adept knowledge, along with a positive attitude and behavior. The study aimed to assess the knowledge, attitude, and perception of Egyptian HCWs and medical students towards human Mpox.

**Methods:**

The present cross-sectional study data was collected from participants between October and December 2022 via a questionnaire. The questionnaire comprised 31 questions in the knowledge section, 11 questions in the attitude section, and 14 in the perception section.

**Results:**

The present study involved a total of 1,034 HCWs and medical students. It was found that 55.3% of the participants demonstrated adequate knowledge about Mpox, whereas 44.5% and 39.8% of the respondents exhibited favorable attitudes and perceptions towards the disease, respectively. Binary logistic regression analysis revealed that adequate knowledge was significantly observed in ages older than 40 years (*p* < 0.001), married participants (*p* < 0.001), and doctors (*p* < 0.001). The positive attitude was significantly observed among the male sex (*p* = 0.045), urban residents (*p* = 0.002), and nurses (*p* = 0.002). Conversely, married participants (p = 0.013), doctors (*p* < 0.001), and individuals employed in pharmacy and laboratory departments (*p* < 0.001) experienced an increase in positive perception.

**Conclusion:**

Knowledge, attitude, and perception towards Mpox among Egyptian HCWs and medical students exhibit suboptimal levels. Addressing these gaps is crucial to controlling and effectively preventing disease transmission.

## 1 Introduction

The monkeypox (Mpox) virus is an enveloped, brick-shaped DNA virus. Two virus clades are identified through genomic sequencing: West African (WA) and Congo Basin (CB) clades. According to virulence, the WA clade is thought to be less virulent and less transmissible than CB (Sale et al., 2006). Although human-to-human transmission is likely, humans are deemed incidental hosts. The Mpox virus can be transmitted to humans via contaminated fomites, respiratory droplets, direct contact with infected animals or humans, ingesting infected meat (Brown and Leggat, 2016), and sexual contact, particularly among gay communities (Amer et al., 2022).

In May 2022, the Mpox disease, previously referred to as monkeypox (World Health Organization [WHO], 2022b), emerged as an epidemic. It infected more than 91,788 confirmed cases and resulted in a total of 167 deaths as of the time of writing this article (WHO, 2023) A total of 89,358 cases were identified in countries and territories where the disease was not previously common, in addition to 1970 cases reported in endemic countries (Centers for Disease Control and Prevention [CDC], 2022a). In September 2022, Egypt officially announced the detection of the first case of Mpox in a 42-year-old man who arrived from Spain. According to WHO Mpox Global Trends, the number of confirmed cases has stood at three until the present (WHO, 2023).

This epidemic began to decline following the provision of vaccination programs and the implementation of relevant infection control (IPC) procedures (WHO, 2023). Notably, this does not imply the conclusion of the epidemic, as there is a possibility of the re-emergence. Although less frequent with DNA viruses, the occurrence of mutation leads to new variants, as was the case of the May outbreak strain (Giorgi et al., 2022). This phenomenon is also observed in other viruses. The lack of understanding of various factors during the initial outbreak in non-endemic countries can contribute to re-emergence of the disease (Amer et al., 2023). Despite the extensive utilization of vaccination, recent studies have reported several breakthrough infections after using the modified vaccinia Ankara–Bavaria Nordic (MVA–BN) vaccine in preventing Mpox infection (Hazra et al., 2022; Bertran et al., 2023; Taha et al., 2023). According to WHO, a contributing factor to the resurgence of Mpox in non-endemic countries is the inadequate knowledge among HCWs. Furthermore, the close contact between HCWs and infected patients increases their risk of contracting the disease (WHO, 2022a). Several reports evaluated the public and HCWs’ knowledge, attitudes, and perceptions (KAP). Studies conducted in Jordan and the Kingdom of Saudi Arabia (KSA) revealed inadequate knowledge regarding Mpox among HCWs and medical students, respectively (Sallam et al., 2022).

For Egypt, a non-endemic country of Mpox, several factors require attention in order to prevent the importation of Mpox. In addition to being a popular tourist destination worldwide, its strategic geographic location in North Africa renders it a convenient host country for African refugees and international students (United Nations High Commissioner for Refugees [UNHCR], 2023; Ghaffar, 2022). Therefore, the cornerstone of preventing the disease, with potential dissemination to other areas of the world, is to ensure that HCWs are equipped with sound knowledge and have positive attitudes and perceptions towards Mpox infection. That applies to medical students recognized for their significant contributions to public health efforts during epidemics (Lazarus et al., 2020; Stachteas et al., 2021). The study aimed to assess the KAP towards Mpox infection among Egyptian HCWs and medical students to increase awareness of Mpox.

## 2 Methods

### 2.1 Study design

This study adopted a cross-sectional design involving HCWs and medical students. The survey tool was developed and pre-tested by reviewing ten HCWs of different positions and by 12 students in different grades. A final questionnaire was generated based on the feedback comments received. Data was gathered from October to December 2022 through the utilization of a Google form, which was subsequently distributed to participants. The sample was selected by a simple random technique with proportional allocation regarding the number of HCWs in each Department at Zagazig University Hospitals in addition to the number of medical students in each academic year at the Faculty of Medicine, Zagazig University, Zagazig, Egypt.

### 2.2 Measures

Regarding the knowledge component, there were a total of 31 questions. Correct answers received a score of 1, do not know was assigned a score of 0, and incorrect answers were assigned a score of -1. The scores were summed to yield a total knowledge score ranging from 0 to 31, where a higher score indicates adequate knowledge when the cut off being at the median of observation.

The attitude component consisted of 11 questions scored on a 3-point Likert scale (disagree, neutral, agree) (Jacoby and Matell, 1971). The responses are 1 for ‘disagree,’ 2 for ‘neutral,’ and 3 for ‘agree.’ Scores were added to yield a total score of 11 to 33 for attitudes, where a higher score denotes a more positive attitude towards the Mpox preventive measures when taken cut off at the median of observation.

The perception component comprised 14 questions, which were evaluated using a 3-point Likert scale, ranging from disagree to neutral to agree. The available responses are as follows: 1 corresponds to ‘disagree,’ 2 corresponds to ‘neutral,’ and 3 corresponds to ‘agree.’ The scores were combined to obtain a total score of 14 to 42 for perception. A higher score indicates a more positive perception towards the Mpox preventive measures, with the cutoff point being the median of observation.

All questions and correct answers to the questionnaire were determined based on guidelines and factsheets for Mpox (CDC, 2022a; CDC, 2022b; CDC, 2022c; WHO, 2022c; WHO, 2022d).

### 2.3 Sample size calculation

The study included a target population of 11,000 HCWs and 7,500 medical students. Assuming that 55% of HCWs and 65% of medical students had adequate knowledge of Mpox, based on recent studies (Alshahrani et al., 2022; Kumar et al., 2023), the minimum sample size to be recruited for the survey from HCWs and medical students at 95% confidence level (CI) and 80% power of the test was 368 and 335, respectively.

Using OpenEpi, Version 3, open-source calculator‐‐SSPropor, the sample size was computed by n = [DEFF*Np (1-p)]/[(d2/Z21-α/2*(N-1) + p*(1-p)], where

n = population size

p = prevalence of adequate knowledge

d = precision

DEFF = design effect

Z1-α/2 = 1.96

### 2.4 Statistical analysis

Data were analyzed using the Statistical Package for the Social Sciences (SPSS) software version 24. Categorical variables were displayed as numbers and percentages (n %) and were compared using Chi-square. In order to identify independent factors correlated with adequate knowledge, positive attitude, and perception towards Mpox. Categorical variables were converted to dummy variables. The category that showed significance in the univariate analysis was assigned a binary value of 1. This value was then used in binary logistic regression analysis and compared to other categories (category) of the same variable, which were assigned a binary value of 0. A probability (*p*) value ≤ 0.05 was considered significant at 95% CI.

## 3 Results

### 3.1 Baseline characteristics of the participants

A total of 1,034 participants, including 646 HCWs and 388 medical students, were interviewed and responded to the questionnaire. More than half of the participants (55.5%) fell within the age range of 20-40 years, with a female predominance (59.6%). Moreover, 73.3% of the survey respondents were from urban areas. The baseline characteristics of the participants are shown in **Table 1**.

**Table 1:** Sociodemographic characteristics of studied group (N =1034).

Characteristics	Categories	Frequency	Percentage	95% CI
Age	< 20 y 20-40 y ≥ 40 y	248 574 212	24.0 55.5 20.5	(21.4- 26.7) (52.4-58.6) (18.1-23.1)
Sex	Male Female	418 616	40.4 59.6	(37.4-43.5) (56.5- 62.6)
Residence	Rural Urban	276 758	26.7 73.3	(24-29.5) (70.5-76.0)
Marital status	Single Married	588 446	56.9 43.1	(53.8-59.9) (40.1-46.2)
Type of HCW	Doctor Nurse Pharmacist Technical Medical students	500 90 40 16 388	48.4 8.7 3.9 1.5 37.5	(45.3-51.5) (7.1-10.6) (2.8-5.2) (0.9-2.5) (34.6-40.6)
Type of department	ICU Infectious General ward Outpatient Pharmacy/laboratory	126 128 316 226 238	12.2 12.4 30.6 21.9 23.0	(10.3-14.3) (10.4-14.5) (27.8-33.5) (19.4-24.5) (20.5-25.5)

CI, confidence interval; HCW, healthcare worker; ICU, intensive care unit.

*Significant difference.

### 3.2 Univariate analysis of the knowledge of study participants

**Figure 1:**
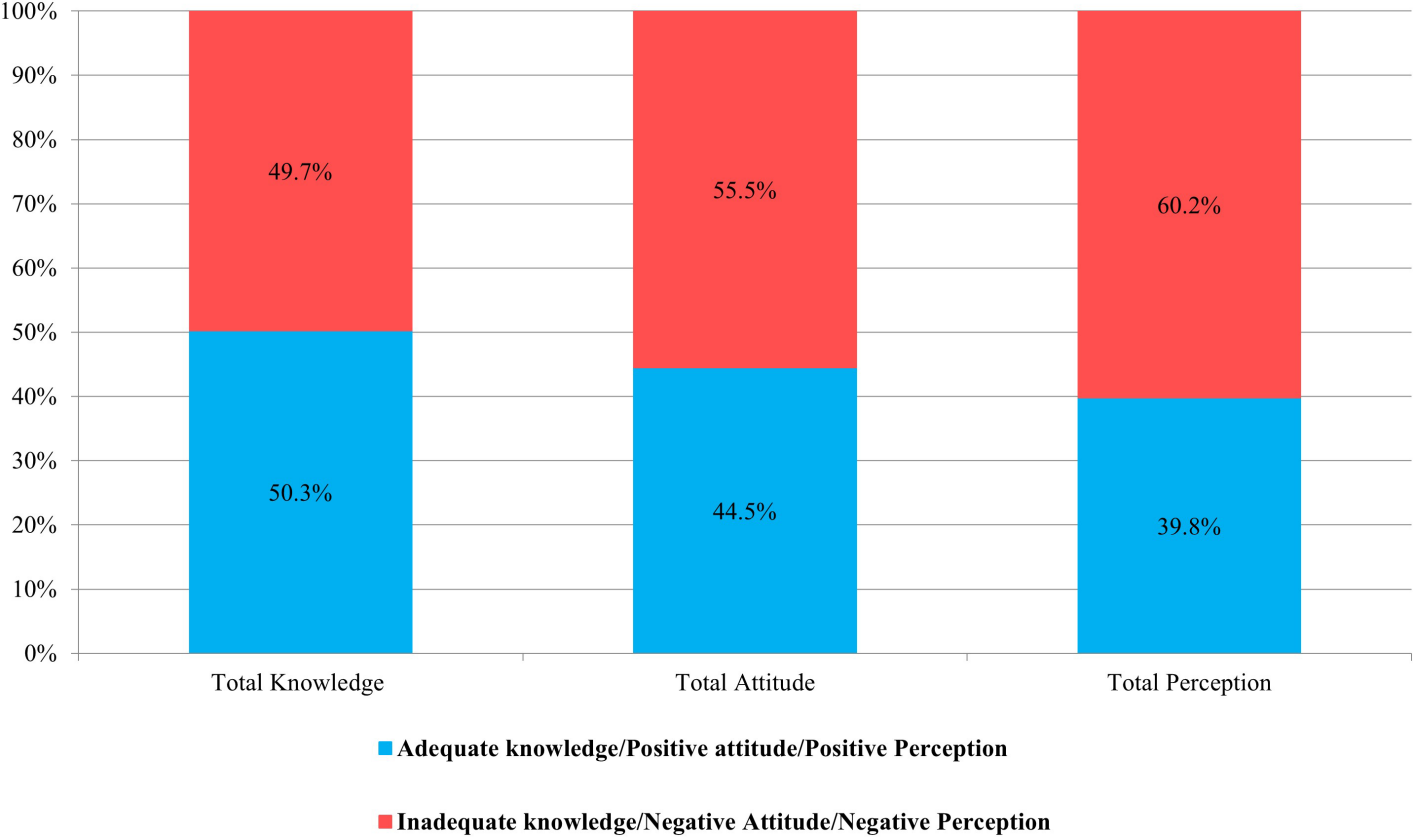
Distribution of overall knowledge, attitude, and perception towards Mpox among study participants.

**Figure 2:**
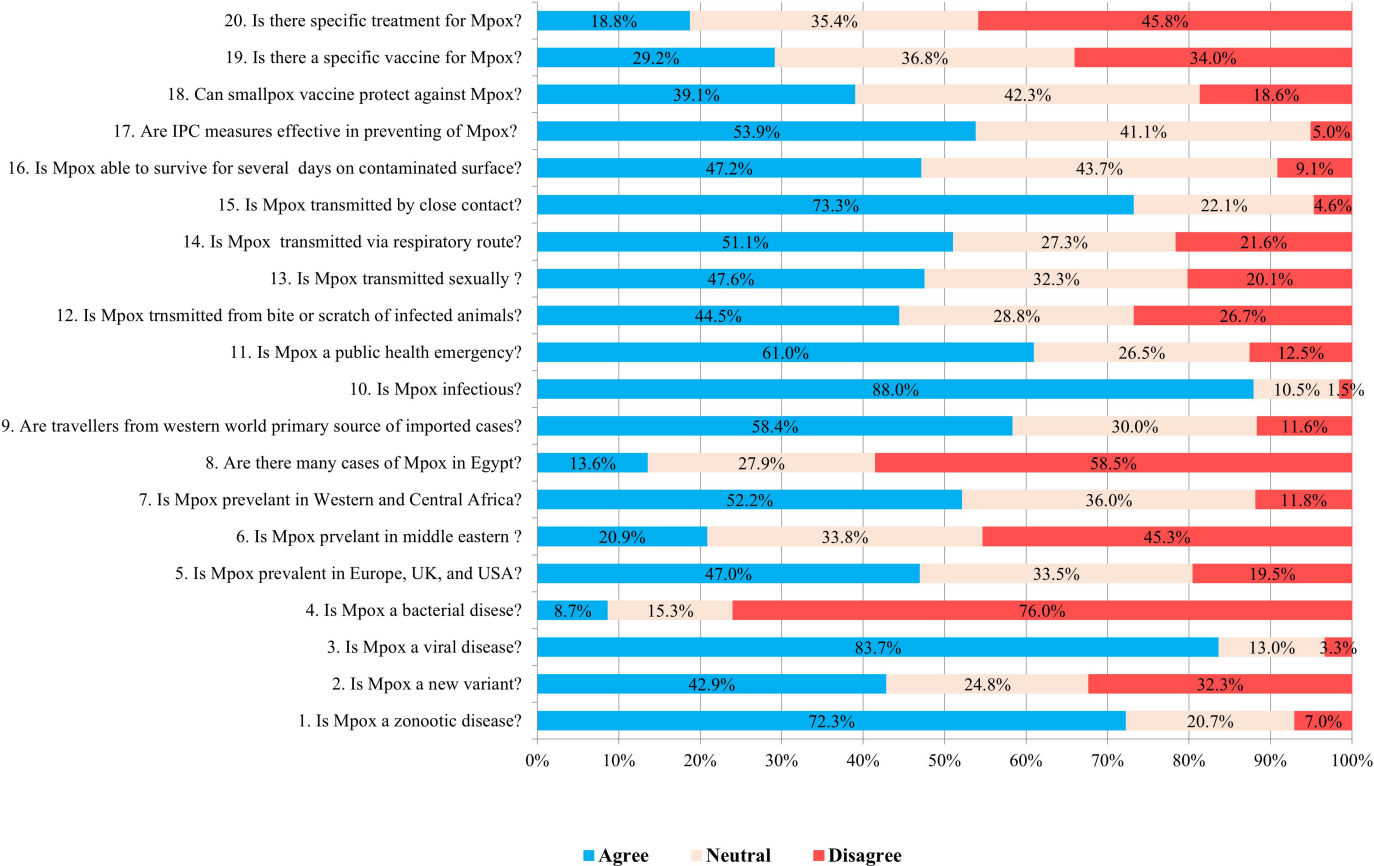
Distribution of responses to microbiology of Mpox (questions 1-4), Mpox epidemiology, (questions 5-8) Mpox transmission (questions 9-16), effectiveness of infection prevention and control (IPC) measures in preventing Mpox (question 17), and Mpox vaccination and treatment among study participants (questions 18-20).

Inadequate knowledge was observed in 49.7% of the participants (**Figure 1**). Participants aged over 40 years old demonstrated a significant level of knowledge (*p* < 0.001) regarding Mpox. Furthermore, married participants and doctors also exhibited a significant level of knowledge about Mpox (*p* < 0.001 and *p* < 0.001, respectively) (**Table 2**). The majority of participants (83.7%) were able to identify Mpox as a zoonotic infectious disease caused by a virus, while a slightly smaller percentage (76%) correctly identified it as not being of bacterial origin. Approximately half of the participants (47.0%) knew that Mpox is prevalent in the Western world. Additionally, more than half of them (52.2%) could recognize that the disease is prevalent in Western and Central Africa, and 58.5% disagreed with its prevalence in Egypt. In addition, 58.4% of participants agreed that Western travelers are the primary source of imported cases. Nearly 61.0% of participants could identify Mpox as a public health hazard. Regarding Mpox transmission routes, close contact transmission was identified by 73.3% of participants, while sexual and respiratory routes were identified by 47.6% and 51.1% of respondents, respectively. In contrast, 53.9% of participants agreed that IPC measures effectively prevent disease. Finally, only 39.1% knew the smallpox vaccine could protect against the disease (**Figure 2**).

**Table 2:** Relation between adequate knowledge, positive attitude and positive perception and participants characteristics (N =1034).

Variable	Adequate knowledge (n=520)		Positive attitude (n=460)		Positive perception (n=412)	
	n (%)	95% CI	n (%)	95% CI	n (%)	95% CI
Age						
< 20 y (n = 248) 20 - < 40 y (n = 574) ≥ 40y (n = 212) ***p* value**	78 (31.5) 300 (52.3) 142 (67.0) < 0.001*	(25.7-37.6) (48.1- 56.4) (60.2-73.3)	102 (41.1%) 260 (45.3%) 98 (46.2%) 0.46	(34.9-47.5) (41.2- 49.5) (39.4-53.2)	82 (33.1%) 212 (36.9%) 118 (55.7%) < 0.001*	(27.2- 39.3) (33.0-41.0) (48.7- 62.5)
Sex						
Male (n = 418) Female (n = 616) ***p* value**	204 (48.8) 316 (51.3) 0.43	(43.9 – 53.7) (47.3-55.3)	202 (48.3) 258 (41.9) 0.04*	(43.4- 53.2) (38.0-45.9)	174 (41.6) 238 (38.6) 0.33	(36.9- 46.5) (34.8-42.6)
Residence						
Rural (n=276) Urban (n=758) ***p* value**	134 (48.6) 386 (50.9) 0.5	(42.5- 54.6) (47.3- 54.5)	104 (37.6) 356 (47.0) 0.007*	(33.6- 41.7) (43.5-50.6)	92 (33.3) 320 (42.2) 0.01*	(27.8-39.2) (38.7-45.8)
Marital status						
Single (n = 588) Married (n = 446) ***p* value**	266 (45.2) 254 (57.0) < 0.001*	(41.2-49.4) (52.2-61.6)	254 (43.2) 206 (46.2) 0.33	(39.2-47.3) (41.5-50.9)	202 (34.4) 210 (47.1) < 0.001*	(30.5-38.3) (42.4- 51.8)
Type of HCW						
Doctor (n = 500) Nurse (n = 90) Pharmacist (n = 40) Technical (n = 16) Medical student (n = 388) ***p* value**	314 (62.8) 40 (44.4) 12 (30.0) 8 (50.0) 146 (37.6) < 0.001*	(58.4-67.0) (34.0-55.3) (16.6-46.5) (24.7-75.3) (32.8-42.7)	224 (44.8) 50 (55.6) 10 (25.0) 6 (37.5) 170 (43.8) 0.02*	(40.4-49.3) (44.7-66.0) (12.7-41.2) (15.2-64.6) (38.8- 48.9)	248 (49.6) 26 (28.9) 12 (30.0) 6 (37.5) 120 (30.9) < 0.001*	(45.1-54.1) (19.8-39.4) (16.6-46.5) (15.2-64.6) (26.4- 35.8)
Type of working department						
ICU (n = 106) Infectious (n = 104) General ward (n = 141) Outpatient (n = 144) Pharmacy/ laboratory (n = 152) Not applicable (n = 387) ***p* value**	52 (49.1) 56 (53.8) 67 (47.5) 75 (52.1) 79 (52.0) 191 (49.4) 0.57	(39.2-59.0) (43.8-63.7) (39.1-56.1) (43.6-60.5) (43.7-60.1) (44.3-54.5)	48 (45.3) 56 (53.8) 50 (35.7) 66 (45.8) 70 (46.1) 170 (43.8) 0.13	(34.7-54.3) (43.8-63.7) (27.6-44.3) (36.6-53.4) (38.2-54.4) (38.7- 49.2)	36 (34.0) 52 (50.0) 52 (37.1) 68 (47.2) 84 (55.3) 120 (30.9) < 0.001*	(30.3-49.6) (42.8-62.4) (29.3-45.7) (39.2-55.8) (47.2- 53.7) (26.0- 36.1)

CI, confidence interval; HCW, healthcare worker; ICU, intensive care unit.

Data were analyzed by Pearson’s chi-square and Fisher’s exact tests when appropriate.

*Significant difference.

### 3.3 Univariate analysis of the attitude of study participants

**Figure 3:**
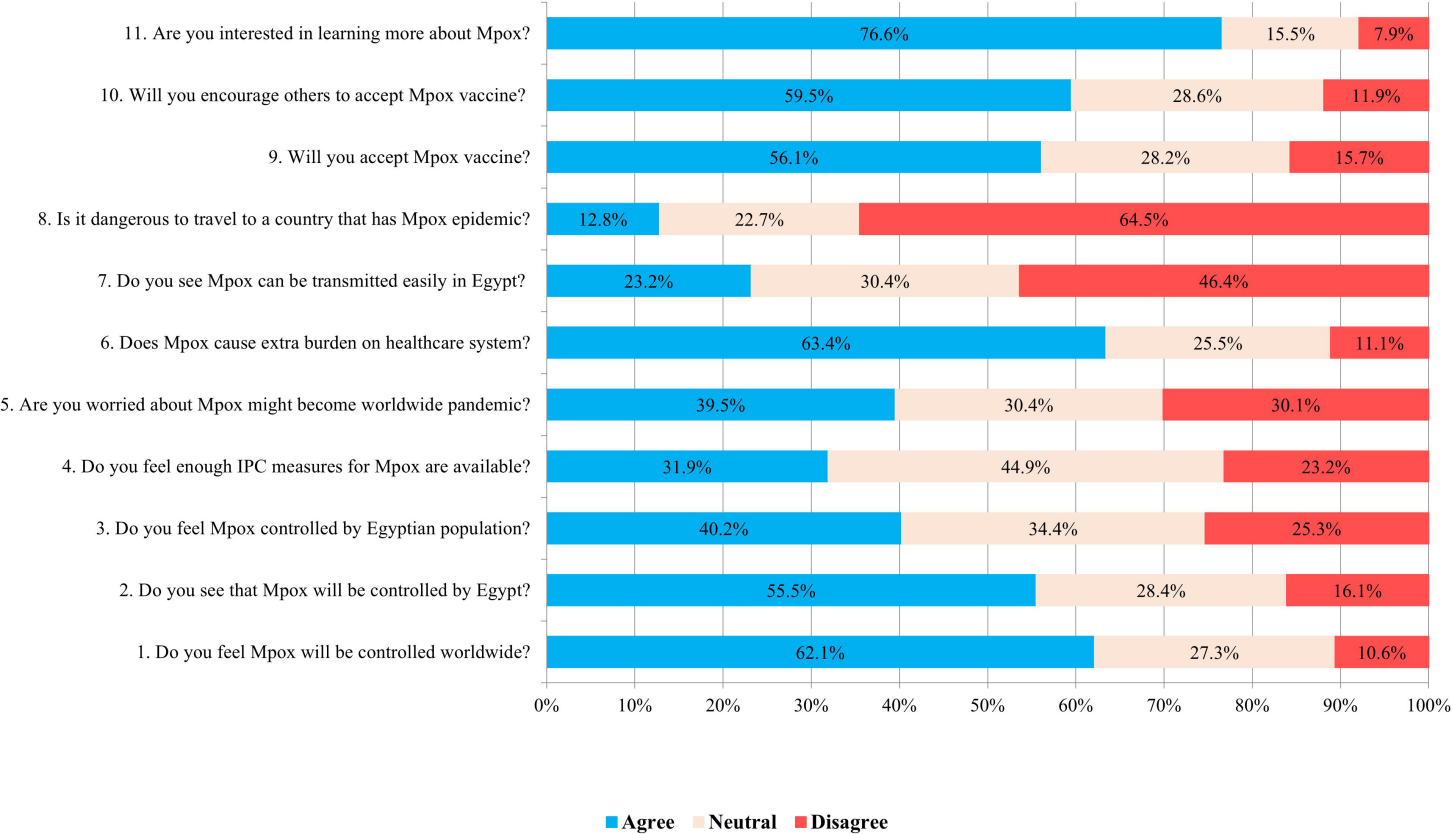
Responses to questions regarding attitude towards Mpox in the studied population.

Overall, 44.5% of participants exhibited a positive attitude toward Mpox (**Figure 1**). A notable prevalence of positive attitude was observed in male participants and individuals residing in urban areas (*p* = 0.04 and 0.007, respectively). Among HCWs, nurses exhibited the highest level of positive attitude (*p* = 0.02) (**Table 2**). Furthermore, 62.1% of participants believed that Mpox would be controlled worldwide. Nevertheless, 63.4% of the participants worried that the disease would burden the healthcare system. Approximately 45.0% were uncertain about the availability of sufficient IPC measures, whereas 46.4% of participants disagreed with the ease of disease transmission in Egypt, while only 12.8% felt the danger of traveling to a country with the Mpox epidemic. Regarding participants’ attitudes towards vaccination, 56.1% of participants expressed their acceptance of the Mpox vaccine, and 59.5% would encourage others to accept it. More than two-thirds of participants (76.6%) displayed their interest in learning more about Mpox (**Figure 3**).

### 3.4 Univariate analysis of the perception of study participants

**Figure 4:**
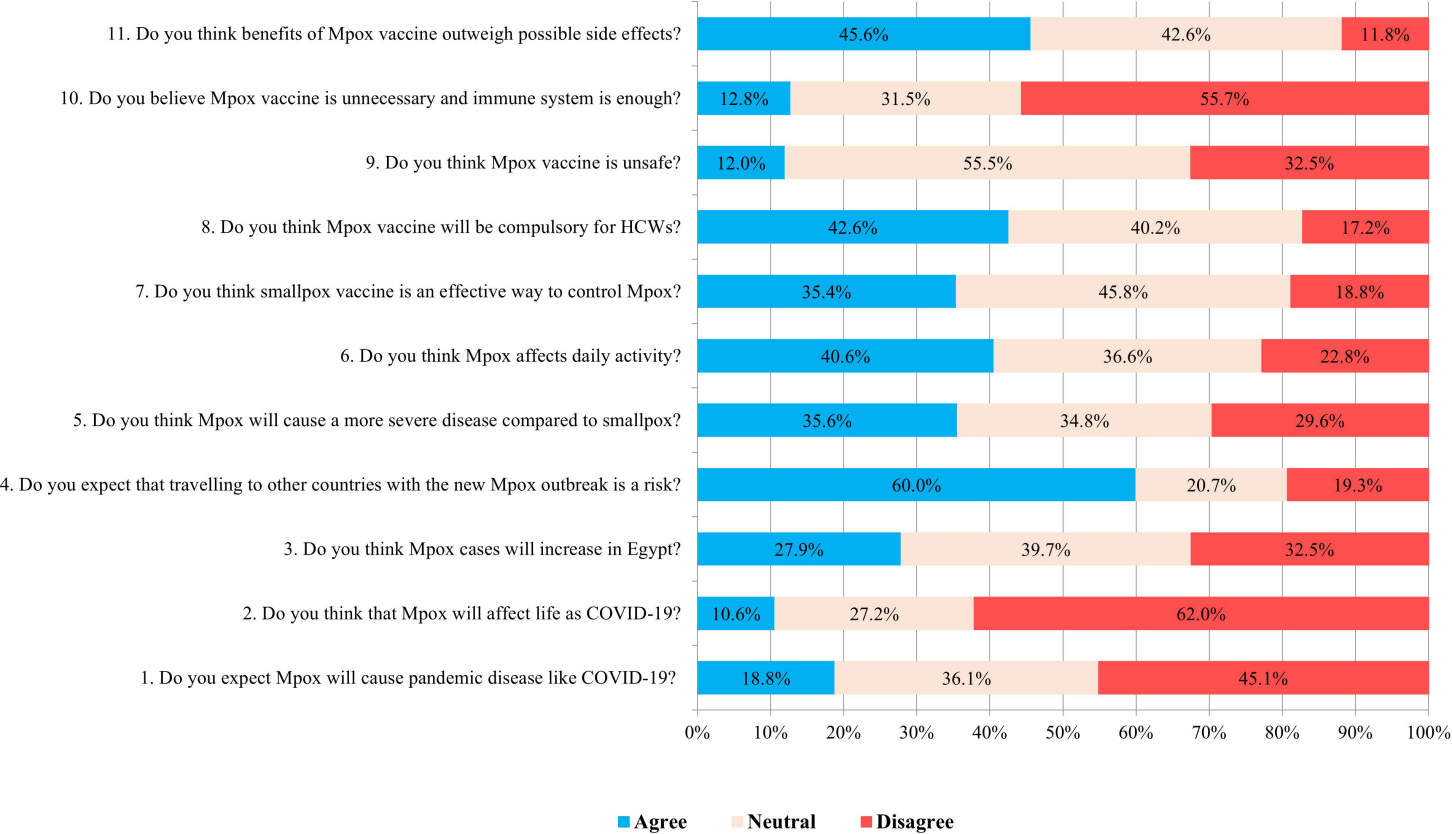
Responses to questions regarding perception towards Mpox in the studied population.

Positive perception towards Mpox was observed in 39.8% of the study participants (**Figure 1**). Participants older than > 40 years, those living in urban localities, married, doctors, and those working in pharmacy/laboratory departments significantly exhibited positive perception (*p* < 0.001, = 0.01, < 0.001, < 0.001, and < 0.001, respectively) (**Table 2**). Among the participants, 18.8% anticipated that Mpox would result in a pandemic similar to COVID-19, whereas only 10.6% believed it would impact life in the same way as COVID-19. Despite participants’ positive attitude (64.5%) towards traveling to a country with the Mpox epidemic, 60.0% still perceive it as a potential hazard. Nevertheless, 45.8% of participants expressed uncertainty regarding the efficacy of the smallpox vaccine in managing Mpox, while 42.6% believed that the vaccine should be mandatory for HCWs. Most participants (55.5%) expressed uncertainty regarding the safety of the Mpox vaccine, although 55.7% acknowledged the necessity of the vaccine and the insufficiency of the immune system alone. Furthermore, 45.6% of the participants concurred that the advantages of the vaccine surpass the potential adverse effects (**Figure 4**).

### 3.5 Predictors of adequate knowledge, positive attitude, and perception in study participants

After including all relevant variables in the binary logistic regression analysis (**Table 3**), the following factors were found to be predictors of adequate knowledge: age older than 40 years (p < 0.001), being married (*p* < 0.001), and being doctors (*p* < 0.001). The factors associated with a positive attitude were being male (*p* = 0.045), living in an urban area (*p* = 0.002), and working as a nurse (*p* = 0.002). In contrast, positive perception was associated with being married (*p* = 0.013), being a doctor (p < 0.001), and working in the pharmacy/laboratory department *(p* < 0.001).

**Table 3:** Binary logistic regression to detect factors affecting knowledge, attitude and perception (N =1034) .

	B	S.E.	P value	OR	95% CI	
					Upper	Lower
**Knowledge**						
Age (≥40 y)	0.86	0.16	< 0.001*	2.383	1.73	3.27
Marital status (Married)	0.471	0.127	< 0.001*	1.60	1.25	2.05
Type of HCW (Doctors)	0.98	0.1 28	< 0.001*	2.68	2.09	3.45
**Attitude**						
Sex (Male)	0.264	0.132	0.045*	1.302	1.006	1.68
Residence (Urban)	0.466	0.147	0.002*	1.59	1.19	2.12
Type of HCW (Nurses)	0. 51	0.24	0.002*	1.08	1.98	2.85
**Perception**						
Age	0.173	0.117	0.139	1.189	.945	1.495
Residence (Urban)	0.096	0.158	0.541	1.101	.808	1.500
Marital status (Married)	0.342	0.138	0.013*	1.408	1.074	1.847
Type of HCW (Doctors)	0.188	0.035	< 0.001*	1.207	1.12	1.448
Working department (Pharmacist /Laboratory)	0.73	0.17	< 0.001*	2.08	1.47	2.95

CI; confidence interval; HCWs, healthcare workers; OR; odds ratio.

*Significant difference.

## 4 Discussion

The present study assessed the knowledge, attitudes, and perceptions of HCWs and medical students regarding Mpox. To our knowledge, few national reports have assessed the knowledge and attitude of Mpox in this specific group (Abd ElHafeez et al., 2023; Alkalash et al., 2023; Swed et al., 2023a; Swed et al., 2023b).

In line with findings from studies conducted in the United States of America (USA) (Bates and Grijalva, 2022), KSA (Alshahrani et al., 2022), Italy (Riccò et al., 2022), and Pakistan (Kumar et al., 2023), the overall knowledge level of the participants was marginally satisfactory. This result is reasonably expected, given the scarcity of cases in Egypt. Furthermore, when comparing the results with reports from the Western world, it is essential to consider factors such as the timing of the survey, sample size, and sociodemographic characteristics of the participants, including age, years of experience, type of healthcare workers, and specialization (Bates and Grijalva, 2022; Riccò et al., 2022). The insights gained from the COVID-19 pandemic highlight the significance of thoroughly understanding all aspects of the disease and taking proactive measures to prepare for the potential occurrence of another surge. The limited awareness among a small group of participants regarding the Mpox “May Epidemic” being referred to a novel Mpox variant could be attributed to the majority of survey respondents being clinicians who received traditional undergraduate medical curriculum. A notable constraint associated with this curriculum format is the tendency for foundational scientific knowledge to be often overlooked upon transitioning to the clinical phase of medical education. Another explanation could be that all released guidelines do not discriminate between the management of Mpox caused by the classical vs. the new variant.

The participants’ apprehension regarding the extensive proliferation of cases in the Western world gives rise to a concerning disparity. There may be a reduced level of suspicion towards travelers from these regions exhibiting potential Mpox symptoms. Moreover, the identification of high-risk groups will aid in preventing the inadvertent omission of potential signs and symptoms that may manifest among particularly susceptible populations (Rodriguez-Morales and Amer, 2022). The fact that the majority of participants acknowledge that the disease can be prevented by implementing effective IPC measures is comforting (Amer et al., 2023). According to a study conducted by Alshahrani et al. in KSA (Alshahrani et al., 2022), the lowest levels of knowledge confronted in this study were those concerning treatment and vaccination. Interestingly, their knowledge levels were even lower than those observed in Turkey (Sahin et al., 2023) and Italy (Riccò et al., 2022). This observation can be attributed to the limited number of cases in Saudi Arabia and Egypt in comparison to Turkey and Italy. The regression analysis conducted in this study yielded contrasting findings compared to a recent Egyptian study (Alkalash et al., 2023) and a KSA report (Alshahrani et al., 2022) but aligns with a study from the Philippines (Berdida, 2023). Our research indicated that individuals above the age of 40 possessed the highest level of knowledge, as supported by statistical analysis. The younger contributors were born after the eradication of smallpox, and there has been a decreasing focus on poxviruses in training and at university-level information. Furthermore, age groups below 40 exhibited a lower number of years of experience in the specific field compared to older age groups. Consistent with previous studies (Depoux et al., 2020; Germani et al., 2022), our study found that married participants exhibited a higher level of knowledge, which may serve as a protective factor against infection transmission to their partners. Our finding that the highest level of knowledge was among doctors is reassuring, which is expected to optimize their capability of managing potential cases.

The overall attitude of participant HCWs and medical students could not be considered convenient. The surveyors expressed doubt regarding the feasibility of disease control among the Egyptian population due to the perceived inadequacy of IPC supplies and subpar hygienic practices. However, it is important to highlight that Egypt has successfully implemented and continues to implement numerous initiatives to enhance the overall sanitation conditions across the country. Although the number of Mpox cases is decreasing, participants’ concern about the disease becoming a global pandemic can be attributed to uncertainty regarding the effectiveness of available vaccines and lack of complete knowledge related to the virus (Hazra et al., 2022; Amer et al., 2023; Bertran et al., 2023). The primary issue most respondents raised is that Mpox imposes an additional financial burden on the affected countries, coinciding with Sahin et al. (Sahin et al., 2023). Our findings emphasize that acceptance of vaccines by HCWs and students is reassuring from the public health perspective for self-protection and for promoting approval in the general population (Maltezou et al., 2019). In response to healthcare workers’ expressed interest in acquiring more knowledge about Mpox, the Egyptian Ministry of Health has issued and actively promoted the Egyptian Guidelines for the Management of Mpox (Today, 2022). Furthermore, Egyptian universities added enough information about Mpox to medical students’ curricula. Our findings in the regression analysis models of the variables’ category most likely associated with positive attitudes are explained as follows: (1) males, may be due to the fear of the misinformation that Mpox is a male-exclusive disease; (2) urban residence; may be due to multiplicity and more accessibility of information sources; (3) nurses; possibly because they are the category of HCWs with more contact with patients.

The overall perception of participants was poor. Misperception of many vaccine-related elements, as reported by other authors (Janssen et al., 2021), can be attributed to emotive and personal factors and obsession as well as other factors (Hazra et al., 2022; Amer et al., 2023; Bertran et al., 2023). The regression analysis revealed a significant level of perception among pharmacists and laboratory workers. Pharmacists are considered the most easily approachable HCWs for offering consultations, especially for individuals lacking adequate healthcare services (Bennett and Goode, 2016; Kassem et al., 2021). In contrast, laboratory HCWs are the healthcare professionals who are most susceptible to infection due to their more frequent and close interactions(Amer et al., 2023).

Contradictory responses were observed in some Knowledge, Attitude, and Perception (KAP) questions during data analysis. When there was adequate knowledge, but negative attitudes and/or perceptions were reported, potential causes may include “social desirability bias” (Betsch and Wicker, 2014) or an “emotional response.” In cases where attitudes scored negative while perceptions scored positively, or vice versa, a more logical comparison or a passionate response was anticipated.

Despite the feasibility and cost-effectiveness of cross-sectional studies, they lack the temporal link between exposure and outcome. The utilization of online questionnaires for data collection can potentially lead to non-response bias and discrepancies in characteristics between respondents and non-respondents. Consequently, to mitigate the impact of these biases, we implemented a prior calculation of sample size, tested and evaluated the survey tool, distributed the questionnaire through various social media platforms, and extended the data collection period. In addition, there was nearly one investigator from each participating department or category of healthcare workers (HCWs) to guarantee that each participant provided precise information, was affiliated with the medical field, and submitted a single response in a timely manner.

## 5 Conclusions

In conclusion, the Mpox-related KAP of Egyptian HCWs and medical students is suboptimal, highlighting the importance of providing suitable information campaigns and educational programs. Medical curricula should be updated to include essential facts about the causative agent and the disease. Moreover, the study findings provide valuable insights into the strengths and weaknesses of Egypt’s healthcare system that need to be judiciously addressed, given its role as a model for other developing countries with limited resources.

## Data availability statement

The raw data supporting the conclusions of this article will be made available by the authors, without undue reservation.

## Ethics statement

The study was conducted according to the guidelines of the Declaration of Helsinki and approved by the Institutional Review Board, Faculty of Medicine, Zagazig University, Egypt (#10577/2/4-2023). Informed consent has been obtained from every study participant.

## Author contributions

FAA: Conceptualization, Investigation, Methodology, Project administration, Supervision, Validation, Writing – original draft, Writing – review & editing. HAN: Data curation, Formal analysis, Investigation, Methodology, Software, Validation, Visualization, Writing – original draft. MG: Investigation, Methodology, Software, Writing – original draft. AMB: Investigation, Resources, Writing – original draft. AAll: Investigation, Writing – review & editing. HK: Investigation, Writing – review & editing. ME: Investigation, Writing – review & editing. HN: Investigation, Writing – review & editing. MS: Investigation, Writing – review & editing. SS: Investigation, Writing – review & editing. AB: Investigation, Writing – review & editing. OA: Investigation, Writing – review & editing. SB: Investigation, Writing – review & editing. AAli: Investigation, Writing – review & editing. FMA: Investigation, Writing – review & editing. AMA: Investigation, Writing – review & editing. NH: Data curation, Formal analysis, Investigation, Methodology, Project administration, Resources, Software, Validation, Visualization, Writing – original draft, Writing – review & editing.
